# Diagnostic étiologique du diabète insipide central: à propos de 41 cas

**DOI:** 10.11604/pamj.2016.24.143.8112

**Published:** 2016-06-14

**Authors:** Fatma Chaker, Melika Chihaoui, Meriem Yazidi, Hedia Slimane

**Affiliations:** 1 Department of Endocrinology and Diabetes, Rabta University Hospital, University of Tunis El Manar, Tunis, Tunisia

**Keywords:** Diabète insipide central, chirurgie, imagerie par résonance magnétique, Central diabetes insipidus, surgery, magnetic resonance imaging

## Abstract

La survenue d'un syndrome polyuro-polydipsiqueavec des urines hypotoniques nécessite une stratégie diagnostique rigoureuse. Le but de cette étude était d’étudier les modalités de diagnostic du diabète insipide central. A travers une étude rétrospective de 41 cas de diabète insipide central(DIC) colligés au service d'Endocrinologie à l'hôpital de la Rabta de Tunis, allant de l'année 1990 à l'an 2013, nous avons relevé les circonstances de découverte du DIC, les anomalies du bilan anté-hypophysaire etde l'imagerie hypophysaire. Le DIC était post opératoire chez 20 patients. La diurèse moyenne de 24 heures était significativement plus élevée chez les patients ayant un DIC en dehors d'un contexte chirurgical. L’épreuve de restriction hydrique était concluante chez tous les patients qui en ont bénéficié. En dehors d'un contexte neurochirurgical, les causes infiltratives étaient retrouvées chez 6 patientset les causes tumorales chez 6 patients. Le DIC était associé à une selle turcique vide dans 1 cas et idiopathique chez 3 malades. L'imagerie par résonnance magnétique hypothalamo-hypophysaire et le bilan anté-hypophysaire sont systématiques en dehors d'un contexte de chirurgie hypophysaire et d'une polydipsie primaire évidente.

## Abstract (en)

The occurrence of polyuria-polydipsia syndrome with hypotonic urine requires careful diagnostic strategy. This study aims to evaluate diagnostic modalities for central diabetes insipidus. We conducted a retrospective study of 41 cases with central diabetes insipidus (CDI). Data were collected at the Department of Endocrinology, University Hospital La Rabta, Tunis, from 1990 to 2013. We identified the circumstances for detecting CDI, the abnormalities in anterior pituitary assessment and pituitary imaging. CDI occurred in the postoperative period in 20 patients. The average urine 24-hour volume was significantly higher in patients with CDI outside a surgical setting. Water deprivation test was successful in all patients who benefited from it. Outside of neurosurgery, infiltration causes were found in 6 patients and tumor causes were found in 6 patients. CDI was associated with empty sella turcica in 1 case and idiopathic sella turcica in 3 patients. Hypothalamic-pituitary magnetic resonance imaging and anterior pituitary balance sheet are systematic outside pituitary surgery setting and obvious primary polydipsia.

## Introduction

Le diabète insipide centralest unemaladie rare due à l'incapacité à retenir l'eau libre par le rein par défaut del'hormone antidiurétique (ADH) [1, 2]. Le diagnostic positif est facile dans un contexte évocateur post chirurgical et tumoral. Par contre, le diagnostic étiologique est moins aisé.En l´absence d´un contexte neurochirurgical, l´IRM occupe actuellement la première ligne pour le diagnostic positif et étiologique du DI central [1]. Cependant, les pathologies infiltratives posent souvent un problème diagnostique et nécessitent d'autres explorations ciblées [1, 3]. Les objectifs de ce travail étaient d’étudier le diagnostic étiologique du diabète insipide central et déterminer les particularités radiologiques selon l’étiologie retenue.

## Méthodes

C'est une étude rétrospective, incluant 41 patients sur une période de 23 ans allant de 1990 à 2013chez qui le diagnostic de DIC a été retenu. Le diabète insipide était évoqué devant une polyurie hypotonique définie par une polyurie de 24h> 3l/24h avec une densité urinaire < ou = à1005 et ou une osmolarité urinaire <300 mosm/l. L'origine centrale du DI était confirmée par l'absence de concentration des urines après la restriction hydrique avec diurèse horaire stable ou augmentée associée à une densité et/ou une osmolarité urinaire stables basses (< 700 mOsm/kg) et par une concentration des urines après l'administration exogène d'AVP avec une densité urinaire à la bandelette supérieure à 1015 et une élévation de l'osmolarité urinaire (<700 mOsm/kg). Nous avons relevé les résultats du bilan anté-hypophysaire et les anomalies radiologiques à l'imagerie hypophysaire.

## Résultats

L’âge moyen des patients était de 35 ans ± 16,4. Trois malades étaient âgés de moins de 10 ans. Le DIC était post opératoire chez 20 patients. Il y avait une différence significative de la diurèse moyenne de 24 heures entre les patients ayant un DIC post opératoire et ceux ayant un DIC en dehors d'un contexte chirurgical (6,1±2,3 versus 8,9±4,5 p=0,02). L’épreuve de restriction hydrique (ERH) a été pratiquée chez 18 patients (45%). Toutes les épreuves faites étaient concluantes. L’épreuve n'a pas été réalisée chez 22 malades dont 17 patients aux antécédents de chirurgie hypophysaire et 5 patients chez qui diurèse de 24h dépassaient 7 litres par 24 heures.

**Figure 1:**
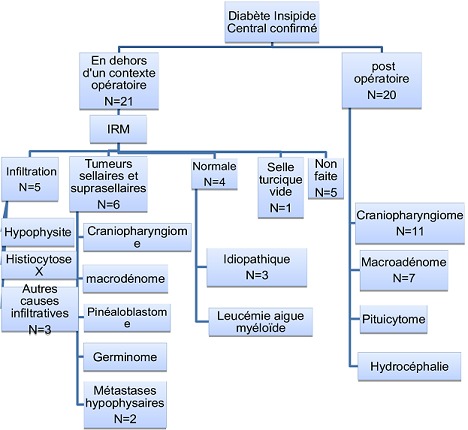
Répartition des différents diagnostics étiologiques du DIC

Chez les 20 patients ayant eu une intervention neurochirurgicale, les indications étaient: un craniopharyngiome chez 11 malades, un adénome hypophysaire chez 7 malades, un pituicytome dans 1 cas et une hydrocéphaliedans un cas (Figure 1). La voie d'abord chirurgicale précisée dans 14 cas était trans-sphénoïdale chez 7 patients et frontale chez 7 patients.

**Figure 2:**
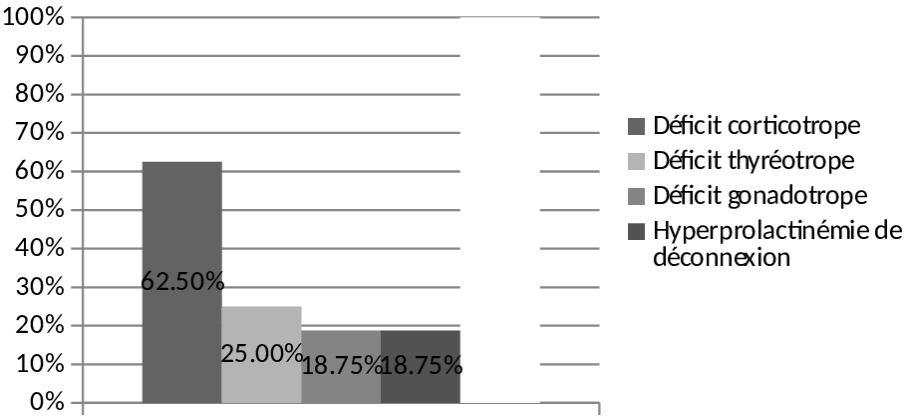
Répartition des malades ayant une insuffisance hypophysaire selon le type de déficit en dehors d'un contexte post opératoire (N=17)

Chez les 21 patients ayant un DIC en dehors d'un contexte post opératoire. Le bilan antéhypophysaire a objectivé un déficit corticotrope dans 62,5% des cas, un déficit thyréotrope dans 25% des cas, un déficit gonadotrope dans 18,75% des cas. Trois patients avaient une hyperprolactinémie de déconnexion (Figure 2).

Une exploration radiologique hypothalamo-hypophysaire s'est basée sur l'IRM hypophysaire mis à part 3 patients explorés par tomodensitométrie hypophysaire dans les années 90 et un malade porteur de prothèse intracardiaque. L'imagerie n'a pas pu être réalisée chez 5 patients qui étaient perdus de vue après la demande de l'IRM.

**Figure 3:**
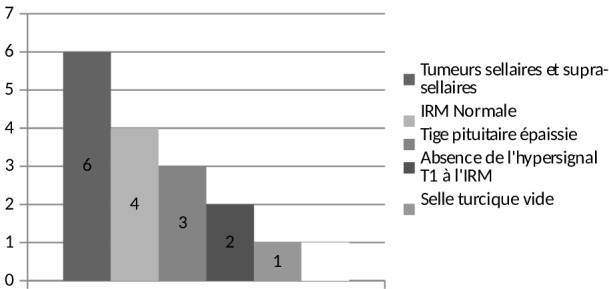
Répartition des malades en dehors d'un contexte opératoire selon les différents aspects radiologiques

Une tumeur de la région hypothalamo-hypophysaire était présente chez 6 malades (33,33%): un pinéaoblastome dans un cas, un germinome hypophysaire dans un cas, un craniopharyngiome dans un cas, un macroadénome hypophysaire dans un cas et enfin des métastases hypophysaires secondaires à un carcinome mammaire dans deux cas (Figure 1). Des anomalies de la tige pituitaire à type d’épaississement étaient relevées chez 5 patients (33,33%) dont 2 cas associé à une perte de l'hypersignal en T1. Un malade avait une selle turcique vide, enfin 4 malades (26,66%) avaient une IRM normale dont un malade porteur de leucémie aigue myéloïde (Figure 3).

Les explorations à visée étiologique chez les patients ayant une infiltration de la tige pituitaire et ou de l'hypophyse ont conclu à une histiocytose X dans un cas, une hypophysite du post partum, dans un cas et une leucémie aigue myéloide dans un cas. Trois patients avaient une tige pituitaire épaissie avec une enquête étiologique négative.

## Discussion

Parmi les 41 patientsinclus, le DIC était post opératoire permanant chez 20 patients. La polyurie hypotonique était le signe révélateur dans tous les cas. En dehors d'un contexte neurochirurgical, les causes infiltratives et tumorales étaient prédominantes. La quantification de la diurèse de 24heures est cependant nécessaire avant d'entamer une enquête étiologique. La polyurie est définie selon divers auteurs par des seuils différents de la diurèse de 24h: supérieure à 2 ml/kg /h [4], supérieure à 30 ml/kg /j [5], supérieure à 2,5L / jour [6, 7], ou supérieure à 250-500 ml/h pendant 2-3 heures consécutives [5, 8]. Une densité urinaire inférieure à 1005 ou une osmolarité urinaire inférieure à 300 voire 250 mOsm/kg sont communément utilisées pour définir le caractère hypotonique des urines [2, 3, 5, 8]. Une osmolarité plasmatique supérieure à 300 mOsm/l en présence d'urine hypotoniques sont également contributifs au diagnostic de DI [4, 5, 7].

Le diagnostic positif du DIC dans la période post-opératoire est posé sur un faisceau d'arguments cliniques et biologiques [9, 10]. L'insuffisance hypophysaire est fréquente [10, 11]. L’épreuve de restriction hydrique est rarement indiquée dans ce contexteet le dosage de l'ADH reste une alternative rarement utilisée [5]. L'IRM hypophysaire postopératoire n'a pas d'intérêt dans le diagnostic du DIC survenu en postopératoire, elle garde ses indications précises selon la pathologie initiale. Chez les patients opérés de notre série, le diagnostic de DIC était retenu devant un SPUPD permanant dans les suites d'une chirurgie de la région hypophysaire et la majorité n'a pas bénéficié d'une ERH.

En dehors d'un contexte post opératoire évident, l'ERH peut trancher d'emblée entre un DI et une polydipsie primitive et dispenser ainsi des explorations ultérieures. Dans notre série, l'ERH était concluante chez tous les patientsqui en ont bénéficié. Cependant, mis à part son côté contraignant pour le patient et le risque de déshydratation [12], l'interprétation de l’épreuve de restriction hydrique reste délicate notamment pour distinguer un DI partiel d'une polydipsie primitive ancienne par diminution du gradient osmolairecorticopapillaire. La réponse rénale à la vasopressine peut également poser un problème diagnostique dans la distinction entre un DIC partiel et un DI néphrogénique avec une résistance incomplète à l'action de l'ADH. La mesure des niveaux de l'ADH plasmatique avant et après l’épreuve de restriction hydrique a été recommandée afin de surmonter les difficultés d'interprétation de l´osmolarité urinaire seule [13, 14]. L'intérêt du dosage de l'ADH est limité par des contraintes méthodologiques comme la clairance rapide [15], l'instabilité pré analytique [16] et la disponibilité de ce dosage dans peu de centres médicaux. En cas de doute persistant, un essai thérapeutique avec de le DDAVP peut être pratiqué sous surveillance stricte clinique et biologique [12].

L'IRM hypophysaire en complément du bilan anté-hypophysaire est actuellement adoptée par plusieurs équipes comme seconde étape après le diagnostic de polyurie hypotonique. Elle permet d’éviter le test de restrictionhydrique dans la moitié des cas [17]. Le DIC apparait lorsque 85% des cellules neurosécrétrices sont détruites. En imagerie cela se traduit par une disparition de l'hyper signal spontané de la post hypophyse en pondération T1 [18, 19]. D'autres anomalies à l'IRM, sans être spécifiques, permettent en plus d'orienter vers une étiologie du DIC, notamment tumorale et infiltrative. Le craniopharyngiome est parmi les tumeurs les plus pourvoyeuses de DIC en raison d'un développement suprasellaire. Cependant, le DIC est rarement le signe révélateur [18]. Les adénomes hypophysaires se compliquent d'un DIC en cas d'apoplexie ou de développement suprasellaire [20]. Les métastases hypophysaires représentent 1% des masses sellaires. Elles sont secondaires aux cancers du sein, comme c’était le cas chez les 2 patients de notre série, aux cancers de l'endomètre, de la prostate, du poumon, du colon et aux mélanomeset le DI est dans 70% des cas le symptôme initial [18, 21]. L’épaississement isolé de la tige pituitaire est une anomalie fréquente au cours du DIC. C'est souvent le contexte clinico biologique et les lésions associées qui orientent le diagnostic étiologique [18, 22]. Les causes infiltratives ou granulomateuses sont les plus fréquentes [12], retrouvées également chez 40% des patients dans notre série. L'infiltration de la tige pituitaire a été rapportée chez 70 à 80% des patients ayant une histiocytose et un diabète insipide central [23]. L'augmentation concomitante de la taille de l'antéhypophyse suggère la présence d'un processus auto-immun [24]. L'incidence du DIC au cours des leucémies aigues est rare. Le mécanisme physiopathologique est une infiltration hypothalamo-hypophysaire par les cellules leucémiques principalement lors de la phase de transformation en leucémie myéloïde aiguë [25]. Les tumeurs germinales du système nerveux central se voient surtout chez l'enfant et l'adolescent, elles sont supra-sellaires dans 40% des cas L'association à une lésion au niveau de la glande pinéale est quasi pathognomonique [22, 26]. A l'heure actuelle, 30% des DIC restent idiopathiques [17, 21]. Cependant, l’évolution fluctuante de l’épaisseur de la tige pituitaire au cours des hypophysites auto-immunes ou dans les germinomes peut faire retenir à tort le diagnostic de diabète insipide idiopathique. Une surveillance régulière est donc indispensable. Si le diabète insipide et la grosse tige pituitaire sont totalement isolés, une simple surveillance, sans preuve histologique, peut être proposée, en renouvelant l'IRM à 3 et à 6 mois, dans l'hypothèse soit d'un germinome qui, en augmentant de taille, fera réaliser une biopsie, soit d'une diminution spontanée de la lésion, très évocatrice alors d'une neuro-hypophysite, soit d'une stabilisation évoquant une histiocytose à évolution prolongée surtout chez les enfants et les adolescents [17].

## Conclusion

L’étude rétrospective des cas de diabète insipide central inclus dans notre série nous a permis d'insister sur les modalités du diagnostic du DIC. Le diagnostic d'une polyurie hypotonique dans les suites d'une intervention sur la région sellaire est suffisant pour retenir l'origine centrale du diabète insipide. En dehors de ce contexte, un test de restriction hydrique permet de différencier d'emblée un diabète insipide d'une polydipsie primaire. L'IRM hypothalamo-hypophysaire et le bilan anté-hypophysaire sont systématiques en dehors d'une polydipsie primaire évidente. En cas d'anomalies hypophysaires à l'IRM, des explorations ciblées permettent d’établie le diagnostic étiologique du DIC. Une surveillance clinique et radiologique peut être proposée en l'absence d'anomalies à l'IRM afin de ne pas retenir à tort le diagnostic de DIC idiopathique.

### Etat des connaissances actuelles sur le sujet

- Le diabète insipide central est défini par une incapacité à retenir l'eau libre qui est due à une libération insuffisante ou inexistante d'hormone antidiurétique (ADH) par l'hypoythalamus. Il s'agit d'une maladie rare avec une prévalence de 1 cas /25000;
- En dehors d'un contexte de chirurgie hypophysaire, le diagnostic du diabète insipide central est plus aisé depuis la généralisation de l'IRM qui peut à la fois faire le diagnostic positif et étiologique.

### Contribution de notre étude à la connaissance

- Il s'agit d'une série de castunisienne d'une maladie rare au sein d'un service d'endocrinologie;
- Les particularités du DIC post opératoire ont été rapportées;
- Les principales étiologies du diabète insipide central en dehors du contexte opératoire ont été discutées.
